# Wildy Prize Lecture, 2020–2021: Who wouldn’t want to discover a new virus?

**DOI:** 10.1099/mic.0.001094

**Published:** 2021-09-01

**Authors:** Graham F. Hatfull

**Affiliations:** ^1^​ Department of Biological Sciences, University of Pittsburgh, Pittsburgh, PA 15260, USA

## Abstract

Innovations in science education are desperately needed to find ways to engage and interest students early in their undergraduate careers. Exposing students to authentic research experiences is highly beneficial, but finding ways to include all types of students and to do this at large scale is especially challenging. An attractive solution is the concept of an inclusive research education community (iREC) in which centralized research leadership and administration supports multiple institutions, including diverse groups of schools and universities, faculty and students. The Science Education Alliance Phage Hunters Advancing Genomics and Evolutionary Sciences (SEA-PHAGES) programme is an excellent example of an iREC, in which students explore viral diversity and evolution through discovery and genomic analysis of novel bacteriophages. The SEA-PHAGES programme has proven to be sustainable, to be implemented at large scale, and to enhance student persistence in science, as well as to produce substantial research advances. Discovering a new virus with the potential for new biological insights and clinical applications is inherently exciting. Who wouldn’t want to discover a new virus?

## Introduction

It was a truly wonderful honour to be selected as the Wildy Prize recipient in 2020. I was especially looking forward to being in Edinburgh and meeting up with friends and colleagues. Alas, COVID hit, the meeting was postponed, and I gave the prize lecture remotely the following year, in April 2021. Isolating and characterizing new viruses is certainly an exciting and revealing endeavour, although SARS-CoV2 is one we could have done without. Here, I’ll try to briefly encapsulate the key messages of the prize lecture I gave at the conference.

I’m sad that I did not knowingly get to meet Dr Wildy, although our paths almost certainly crossed in the 1980s, when I was a graduate student with Willie Donachie at Edinburgh University and attended several memorable Society for General Microbiology general meetings; Peter Wildy was SGM President at that time. Dr Wildy hailed from Tunbridge Wells, just down the road from Maidstone, Kent where I spent my early years, and his commitment to science communication and education were hallmarks of his career and the focus of the prize named for him. I’ll recount our forays into bacteriophage discovery and genomics and the role they play in advancing both microbiology and science education.

## Challenges in science education

The challenges in science education are numerous and complex but are reflected in our scientific communities through limited inclusion, restricted diversity, and homogeneity of experiences and backgrounds [1]. Diversity is not a nicety but an essential part of doing science, as progress requires different ways of thinking and new approaches to old problems [2]. And yet in the USA, student persistence in scientific training at the undergraduate level is atrocious, with only about 40 % of students entering as science majors completing degrees in science [3]. It is about half that for underrepresented minority students. Why are we so bad at inspiring students to be scientists? What are we doing wrong?

The advantages of student engagement in research are well documented, and a hopeful antidote to persistent woes [4]. However, the number of students engaging in apprentice-like research experiences is obviously quite limited. There are only so many faculty, research labs and senior researcher mentors to accommodate a very small minority of all students in science programmes [5]. Because the opportunities are tightly constrained, access is tightly limited, and as most students don’t have prior research experiences, selection is typically based on academic performance in classroom studies. In the absence of other data, this is perhaps understandable, but it simply amplifies the biases in educational access from a very early age, shaped by demographic and economic disparities. Moreover, the research experiences are often reserved for more senior students, who have established an academic record on which they can be evaluated.

## Re-engineering the problem

**Fig. 1.**
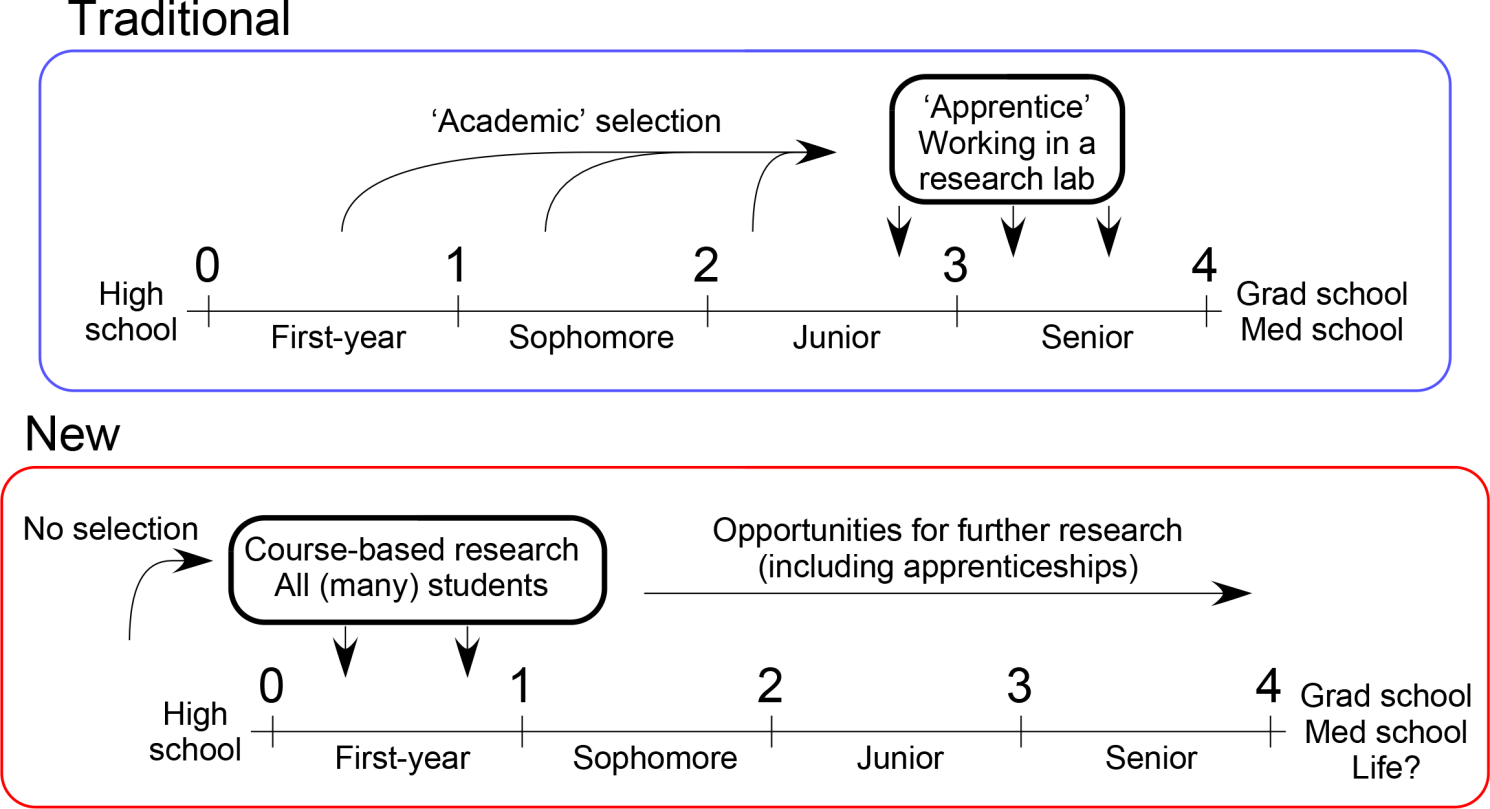
Rethinking engagement of undergraduate students in research. In a more traditional system for engaging students in research (blue box), students work in an apprentice capacity in a research lab, trained by graduate students or postdoctoral researchers, during the later college years. These students are often selected based on their academic coursework performance and are mostly headed to graduate or medical school. In a newer configuration (red box), students take a research-based course early in their college careers, and these courses are open to many students without selection on academic coursework. Such experiences provide a foundation on which students can build in their later college years. These experiences not only benefit those destined for graduate or medical school, but all walks of life.

An alternative way of thinking about the issue is to find ways for students to engage in research early in their scientific programmes, and to do so in an inclusive and scalable way (Fig. 1). If students can start doing research in their first year of college/university, then it provides an opportunity to engage and excite students about doing science, and it prepares them for their future studies, including research. This is a fine idea, but what kind of research project is suitable for first-year students, and how can this be done at large scale; not just in one institution, but in many? And how can institutions implement any of this if they do not have a robust research infrastructure? These questions are at the heart of the general concept of an inclusive research education community (iREC), and bacteriophage discovery and genomics is an especially appropriate scientific focus [6].

## An inclusive research education community (iREC)

**Fig. 2.**
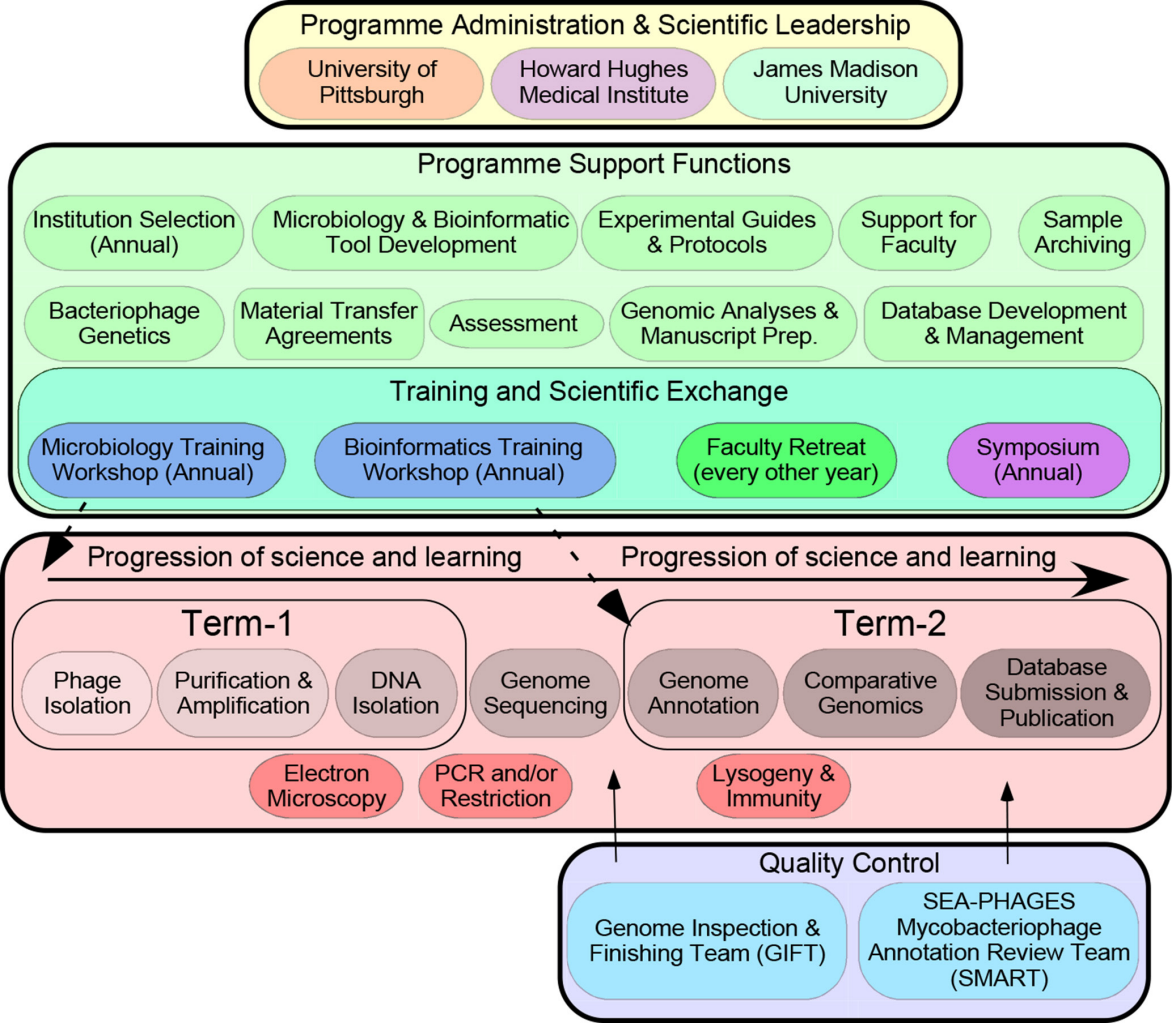
Organization and structure of the Science Education Alliance Phage Hunters Advancing Genomics and Evolutionary Sciences (SEA-PHAGES) programme. SEA-PHAGES programme administrators (yellow box, top) oversee support components critical to programme implementation (green box, upper middle). The typical two-term course structure (pink box, lower middle) includes phage isolation through comparative genomics; additional characterization includes electron microscopy and PCR/restriction analysis. Sequence and annotation quality control are shared with SEA-PHAGES faculty teams. Reproduced with permission from reference [20].

The core concept of an iREC is a unified programmatic infrastructure with centralized administration, scientific coordination, training and resources [6] (Fig. 2). This infrastructure supports a large number of participating institutions, each of which teaches a research-based course. Participation is inclusive, and institutions may be community colleges or polytechnics with few research opportunities, or research-intensive universities. The faculty assigned to teach the research course do not need to have expertise in the scientific topic, as training, resources and scientific support are provided centrally. The community formed through the network of participating schools and faculty brings its own benefits and an abundance of collaborative opportunities. The iREC structure is a general one that can accommodate many different scientific questions, and the Genomics Education Partnership (GEP) [7] and Tiny Earth (https://tinyearth.wisc.edu/) initiatives are two examples. A potential downside of the iREC model is that funds are required to support the central structure, and such funds can be difficult to acquire [6]. Nonetheless, the iREC structure has the capacity to include a very large number of students, and thus the per-student cost can be very low, making it economically efficient. Furthermore, the gains in terms of both research and education can be substantial, giving an overall good cost–benefit ratio.

## Science Education Alliance Phage Hunters Advancing Genomics and Evolutionary Sciences (SEA-PHAGES)

**Fig. 3.**
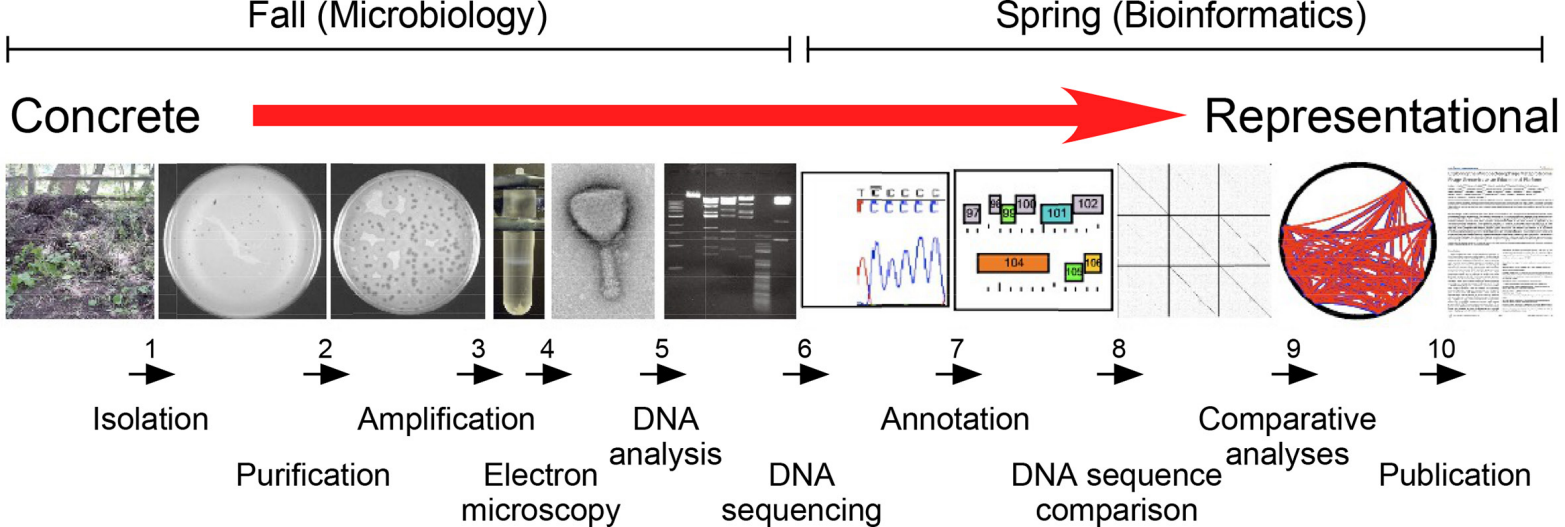
A general platform for phage discovery and genomics. The process of phage discovery can be described in 10 steps, from isolation and purification through to annotation and comparative genomic analyses. These begin with relatively simple and conceptually concrete processes that do not require prior research expertise or any particular skill. However, they progress through a series of more representational processes, including the conceptually abstract process of genome annotation. In the SEA-PHAGES programme, this is typically implemented over two college terms, with the microbiology taught in the fall term, and the bioinformatics in the spring term. However, there is considerable flexibility within the overall platform, and it can be taught with the two terms switched, as an open-ended apprentice-like experience, as an intense 2-week workshop, or extended over several years.

SEA-PHAGES is the largest and arguably the highest impact of all iRECs developed to date [6, 8, 9]. The programme was started in 2008 but emerged from the Phage Hunters Integrating Research and Education (PHIRE) programme developed previously at the University of Pittsburgh, USA [10]. The core scientific platform (Fig. 3) is the same in both programmes and focuses on the discovery of new bacteriophages and their genomic characterization with a view to understanding viral diversity and the evolutionary processes giving rise to the viral population [11]. Students start with an environmental sample (soil or compost is common) and after a brief extraction, mix this with a bacterial culture and plate out on solid media to look for plaques. A student can then purify and amplify their newly isolated phage, name it, isolate genomic DNA, sequence it, bioinformatically annotate the genome, and compare it to other known phage genomes. The techniques are relatively simple, such that no prior research experience is needed, and there is no need to select students based on spurious criteria – this is something that everyone can do. Isolating a new virus and characterizing it is cool. Who wouldn’t want to discover a new virus?

There is an important underpinning to the scientific rationale to this approach. Our phage ecologist colleagues have shown that the bacteriophage population at large is vast (a total of 10^31^ phage particles), dynamic (10^23^ infections per second) and old, perhaps dating back to the early days of microbial life [12, 13]. Not surprisingly, the phage population is enormously diverse, with many different types, and many different genomes and genes [14, 15]. Given their numbers and diversity, phage genomes are clearly the largest unexplored reservoir of sequence information in the biosphere. As genetic expeditioners, don’t you want to know what all that stuff is? Moreover, bacteriophages have played critical roles in scientific advancement. They were essential for the emergence of the discipline of molecular biology and fuelled two major biotechnological revolutions with the use of restriction enzymes for gene cloning and CRISPR–Cas for genome engineering [16]. And phages still hold considerable potential as clinical tools, including as antibacterial therapeutics.

Because of overall phage diversity, students have excellent prospects of isolating a phage that has not been previously reported, naming it, and having the findings contribute to our understanding of viral biology. This is important not only for the scientific gains, but for educational advancement too. The discovery process contributes to a high level of project ownership, in which students recognize that they are doing something important, and they are making a personal contribution to it [17–19]. Being able to name the phages adds strongly to this sense of project ownership, while also reflecting the individual variation seen in phage genomes that defies more systematic organization.

The SEA-PHAGES programme began with 12 participating schools, but additional schools have joined the programme each year, and currently there are 165 institutions, with about 5500 student phage hunters annually [6]. Most of the schools are within the continental USA, but there are participants from Canada, New Zealand, Nigeria, Mexico and Puerto Rico. The institutions are well distributed across the wide range of Carnegie classifications [6], with 20 % being high research (R1) universities, 50 % lower research activity and master’s institutions, and 30 % non-research colleges (including community and tribal colleges). In total, nearly 40 000 students have been phage hunters in the SEA-PHAGES programme.

What do students do in the SEA-PHAGES programme? Typically, there are two courses, one spanning each of the fall and spring terms, with the first term involving the microbiological wet lab work of phage isolation, purification, amplification etc., and the second focusing on bioinformatics, genome annotations and comparative genomic analyses [20]. Phage DNA are usually shipped to the University of Pittsburgh late in the fall term, and sequenced in the November–January interval, with sequences returned to the schools at the beginning of the spring term. All phage data are organized and stored in the PhagesDB database [21] and are accessible to all students at the website https://phagesdb.org. Students spend about 4 h per week and typically enrol in their first year at college (although some do so in the second or later years). Usually, the SEA-PHAGES courses fit into the curriculum as replacements for introductory biology laboratories that use a more traditional and non-research syllabus.

## Advances in understanding phage diversity

Because of the specificity of particular phages for their bacterial hosts, the choice of bacterial strains for phage isolation is critical. Many years ago, we chose to use *Mycobacterium smegmatis* mc^2^155 as a phage host, as it is a relatively fast-growing non-pathogenic relative of human pathogens such as *Mycobacterium tuberculosis* and *Mycobacterium abscessus* [22]. *M. smegmatis* has been used for the vast majority of the phage isolates, although the SEA-PHAGES programme has used several other strains within the phylum Actinobacteria, including *Corynebacterium *, *Gordonia *, *Streptomyces *, *Arthrobacter *, *Rhodococcus* and *Microbacterium* [20]. Altogether, these strains span 10 genera, 70 different species and 107 different strains. The focus on closely related host strains is deliberate and based on the idea that the phages of related hosts are more likely to share genetic information. Thus, genomic comparisons can provide insights into how phages migrate across microbial genetic landscapes, exchanging genes along the way.

The collection of phages on these strains has grown to nearly 19 000, of which over 3700 are completely sequenced and annotated. About 10 000 of the phages were isolated on *M. smegmatis* and 2000 of these are sequenced; there are smaller collections on the other bacterial hosts, and all the data are available at https://phagesdb.org. The PhagesDB database [21] is also linked to a second database used by the Phamerator package [23], which provides several key functionalities, including genome comparisons. A software package ‘pdm_utils’ provides the tools for coordinating data between these and GenBank, and for extracting data [24].

The substantial diversity of these phages presents some challenges in organizing them in helpful ways [25]. There are only a couple of instances of identical phages being isolated from different places at different times (but several instances of the same phage arising intra-lab from contamination events). However, when phages are compared pairwise, there are numerous ways in which they can differ. They may vary by just a few single-nucleotide polymorphisms (SNPs) or they may have identical sequences but one phage with more or fewer genes than the other. They may differ by just a few genes or they may have extensive DNA similarity over half the genome and none across the other half; or they may share little or no sequence similarity at all at either the DNA or protein levels; and everything in between! For convenience, phages are assigned to ‘clusters’, such that phages in different clusters share little if any sequence similarity [25]. Initially, a cutoff value of DNA sequence similarity spanning 50 % of the genome lengths was used to place two phages in the same cluster, although this has since been revised to a threshold value of an average of 35 % shared gene content [26]. However, this is essentially an arbitrary value and subject to further revision. Some clusters have evident substructure when compared using average nucleotide identity (ANI) and can be divided into ‘subclusters’. Some phages have no close relatives and are referred to as ‘singletons’. These assignments evolve as the databases grow, especially as new relatives of extant singletons are identified, which now form new clusters. Clusters are designated Clusters A, B, C etc., and subclusters are denoted Subclusters A1, A2, A3, etc. Clusters commonly contain phages of only a single host genus, and allocations of ‘naming space’ (e.g. Cluster EA–EM, GA–GM) are pre-assigned to phages isolated on a particular host genus (in this example, *Microbacterium *) [20].

Currently, the dataset contains 138 clusters and 64 singletons, such that there are over 200 distinct ‘types’ of phages that are different from each other and share little genetic information. Thus, on average, there are about 10 representatives of each type, but the distribution is highly variable. For example, at one extreme there are the 64 singletons with only 1 example of each, by definition, and at the other extreme there are over 680 Cluster A phages (although these are divided into 20 subclusters). In general, a cluster contains phages isolated on a single host genus, but there are some exceptions, notably within Cluster A, in which all of the subclusters are *Mycobacterium* phages, with the exception of subcluster A15, which has *Gordonia* phages [20]. Moreover, analysis by gene content comparison shows that there are some parts of ‘genome sequence space’, where phages of different host genera are more closely related than others [20].

**Fig. 4.**
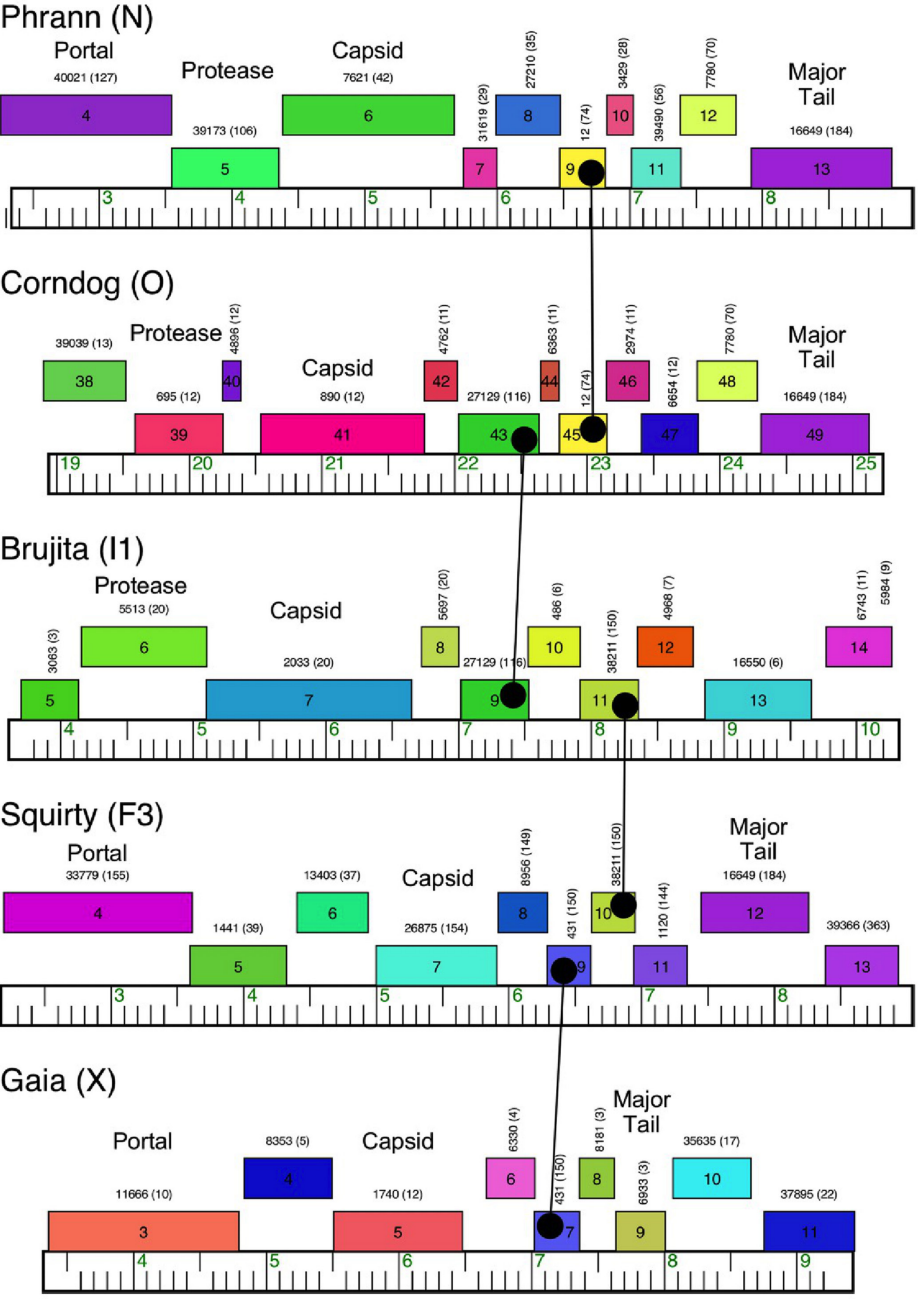
Phage genomic mosaicism. Short segments (~6 kbp) of five phages are shown that are unrelated to each other at the DNA sequence level. Above each genome (with kbp markers) are shown predicted genes in coloured boxes with the gene names within each box. The genes are colour coordinated according to their amino acid sequence relationships, with closely related genes in the same colour. Above each gene box is the number of the phamily (pham) to which the gene is assigned, with the number of pham members shown in parentheses. There are numerous examples where two genomes (e.g. Phrann and Corndog) have related genes (e.g. Phrann *9* and Corndog *45*, related to each other and assigned to the same pham, pham 12) that have diverged from a common ancestor (indicated by the lines ending in circles). However, these sit in distinct genomic contexts in which the flanking genes to the left and to the right are completely different. Other examples of this single-gene mosaicism are shown.

The most evident architectural feature of phage genomes is that they are pervasively mosaic [27]. As such, each genome can be thought of as an assembly of individual units (or ‘mosaic tiles’), each of which can be just a single gene (Fig. 4) [28]. The vast array of different genes can potentially be assembled in a huge number of combinations, in large part accounting for the great diversity that is observed [27]. In practice, this can be seen by comparing genomes that are otherwise not closely related at the DNA sequence level but which share common genes (with shared amino acid sequences) derived from a common ancestor (Fig. 4). However, the adjacent genes are quite different, such that the two related genes are sitting in completely different genomic contexts (Fig. 4). How these mosaic structures are formed remains unclear, but they likely arise through illegitimate recombination events (or replication errors) that require little or no DNA sequence homology [29].

## The endless arms race

**Fig. 5.**
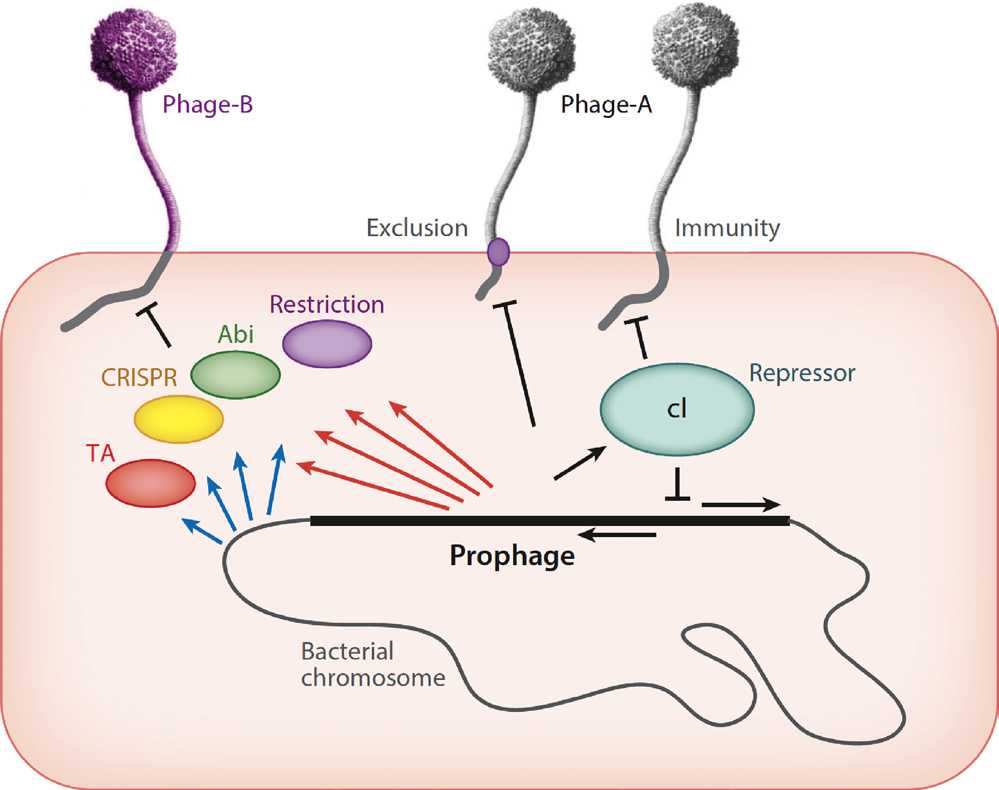
Phage–host dynamics. A lysogenic cell is depicted carrying a prophage integrated into the bacterial chromosome (not to scale). The prophage genome is derived from phage A and encodes a repressor protein (cI) that shuts down lytic genes of both the integrated prophage and superinfecting phage A particles. Some prophages may express membrane proteins that prevent superinfection by the same phage (phage A) or closely related phages. The bacterial chromosome may express a variety of systems to defend against viral attack (blue arrows), including restriction, various abortive infection (abi), CRISPR–Cas and toxin–antitoxin (TA) systems. Prophages can express analogous systems (red arrows) that defend against infection by heterotypic (i.e. unrelated) phages, such as phage B. Reproduced with permission from reference [20].

Phage diversity is largely driven by the ongoing battle between bacteria and bacteriophages, in which the bacteria are constantly striving to survive lytic infections, and the phages are finding bacteria in which they can replicate [30]. But phages are also constantly competing against each other, and the mechanisms involved are less well explored. This competition can be exerted by prophages that collude with their bacterial hosts to defend against infection of other unrelated (i.e. heterotypic) phages, but also to prevent other phages from infecting when a phage is growing lytically (Fig. 5).

There are a growing number of examples among the actinobacteriophages of this first scenario [31–33]. Discovering these systems is a direct benefit of having large collections of individual phages isolated on a single common bacterial host strain. Temperate phages can be used to readily construct lysogenic strains, and then a panel of phages can be tested for their efficiencies of plaquing (eop) on the lysogen relative to the non-lysogen parent strain. When eop differences are observed, they arise either because the superinfecting phage and the lysogens are homoimmune (which is common if the phages are closely related or within the same subcluster), or because the prophage expresses a defence mechanism that prevents productive infection of a second unrelated phage (Fig. 5). These prophage-mediated defence systems are often surprisingly specific for the attacking phage. The specific prophage genes involved can be determined by constructing mutant strains in which genes have been deleted from the prophage, by constructing recombinant strains expressing candidate prophage genes and by RNAseq analysis showing which prophage genes are lysogenically expressed [31]. Although not all temperate phages carry such defence genes, many do, and there is an abundance of defence systems awaiting discovery.

Just as phages carry genes to counteract host defences, such as restriction or CRISPR–Cas [34, 35], phages also carry counter-defence systems that neutralize prophage-mediated defences. An intriguing example is gene *54* in phage Tweety, which antagonizes defences encoded by both Phrann and MichelleMyBell prophages [31]. Phrann and MichelleMyBell prophages both defend against Tweety, reducing eop by four–five orders of magnitude. However, defence escape mutants can be readily isolated with alternations in a tetrapeptide repeat region in the middle of the *54* gene product (gp54). These escape mutants typically have either fewer or more repeat copies than the parent, and appear to ‘tune’ the counter-defence such that it operates against either Phrann or MicheleMyBell defences, specifically [31]. The mechanism by which this works remains obscure.

Phages grow lytically in natural environments where other phages might try to crash the party and replicate in the same cell, reducing the production of the phage that got there first. Phages can counteract this by excluding entry of the competing phage. An interesting example is revealed by phage Fruitloop, which expresses an early lytic protein, gp52, that interacts directly with and inactivates the host DivIVA (Wag31) protein [36]. Other phages, such as Rosebush, are dependent on DivIVA for infection, and thus are unable to infect a Fruitloop-infected cell. This process was discovered by a screen for phage proteins that are toxic when expressed in bacterial cells, and the identification of its interacting host partner protein, and illustrates the broad utility of this strategy [37–39].

## Mycobacteriophage-based genetic tools

A key advantage in exploring mycobacteriophages is that they provide a powerful toolbox for advancing mycobacterial genetics. This would be true for any phage–host combination, but is especially pertinent because of the challenges in working with *M. tuberculosis *, which is not only virulent, but grows extremely slowly (24 h doubling rate). Many genetic tools have been developed and are widely used in mycobacterial genetics. These include integration-proficient plasmid vectors using phage-encoded integration systems to promote transformation [40–43], repressor-based selectable markers [44, 45], phage-derived origins of replication [46], recombineering for gene knockouts and mutant construction [47–50], and phage delivery systems for transposons, allelic exchange substrates and reporter genes [51–54]. The recombineering systems are also important for engineering of the phages themselves [55–57], especially when combined with CRISPR–Cas counter-selection [58]. There are likely many more applications yet to be developed from these phages.

## Therapeutic potential of mycobacteriophages

If you isolated a new virus, wouldn’t it be neat if it was not only a useful source of new biological insights but also useful clinically? Our first foray into the potential therapeutic use of phages was in response to a request from our colleagues at the Great Ormond Street Hospital in London, UK [59]. Two young cystic fibrosis patients suffering from disseminated drug-resistant *Mycobacterium abscessus* infections following bilateral lung transplants were out of treatment options, and therapeutic use of phages was contemplated. Because few, if any, phages of *M. abscessus* had been described previously, we screened a subset of the *M. smegmatis* phages to find those that infect these two particular strains of *M. abscessus *. The subset of phages screened were selected from the genomic relationships, and from what we knew about their infection of other mycobacterial hosts [60]. For one of the strains, we were unsuccessful, and the patient passed away within a couple of months. For the second patient, we identified several useful phages, and assembled a cocktail of three phages, two of which were engineered derivatives of temperate phages that grow lytically [55, 59]. With regulatory approval for compassionate use, the phages were administered intravenously at a dosage of each at 10^9^ plaque-forming units (p.f.u.), twice daily. No serious adverse reactions were observed, and the patient saw substantial resolution of the infection over a course of several weeks [59]. The patient was able to return to a normal life, although the longer-term prospects are unclear. The question now is whether this success can be translated into other patients with similar *M. abscessus* infections. We are finding that there is enormous variation among *M. abscessus* clinical isolates in their phage infection profiles, and it will require considerable effort to assemble a phage cocktail that acts broadly across this strain variation [61, 62]. Nonetheless, if phages can be matched to a patient’s bacterial isolate, the prospects of providing substantial clinical benefits are considerable.

Although there is much to learn about the therapeutic potential of the mycobacteriophages, a number of patients have now shown benefits from this approach, and these would not have been helped without the great phage collection assembled by the SEA-PHAGES programme. At this stage, it is simply not predictable which phages will turn out to be useful therapeutically, but each student knows that their efforts may contribute to the finding of therapeutic solutions.

## Prospects for further iREC development

The SEA-PHAGES programme represents an excellent example of an iREC, but what are the prospects for the development of additional programmes based on other scientific questions and topics? We noted previously seven helpful project attributes for designing integrated research–education programmes, and these are generally applicable to other iREC programmes (Table 1) [11]. To target novice researchers, it is important that there is technical and conceptual simplicity (attributes #1 and #2), and a parallel project structure (attribute #5) facilitates implementation at large scale. If the research is authentic – including discovery-based approaches – then this adds relevance and student motivation (attribute #6), and student engagement is enhanced by the sense that what they do is important to them (attribute #7). It is also helpful if there’s project flexibility that eases scheduling constraints (attribute #3) and multiple milestones help to avoid a sense of failure if students don’t accomplish all the project goals (attribute #4). In general, these align well with facets of the Persistence In The Sciences (PITS) assessment instrument [19] including project ownership, self-efficacy, science identity, scientific community values, networking and future intent, adding strength to these as potential iREC design features.

**Table 1.** Helpful attributes of an iREC programme

Attribute	Description
1	Technical simplicity, especially at initial stages
2	Conceptual simplicity and minimal background requirements
3	Compatibility with flexible scheduling
4	Multiple achievement milestones
5	Parallel project structure enabling large numbers of students
6	Authentic research contributing to peer-reviewed literature
7	Project ownership

Adapted from ref. [11].

It is also helpful for an iREC to be led by an established research group that provides scientific leadership. Financial support for the programme leadership activities – including training, workshops, databases and resources such as protocols – is essential, and can be challenging to procure. Nonetheless, the rationale for this support is strong, and includes the low per-student costs when the programme runs at large scale, and the substantial research and educational advances achievable with large-scale implementation [6]. Finally, we note that even though iREC programmes may start out fairly small, they can expand quite quickly if the programme infrastructure is robust. In anticipation of this, it is useful if systems for handling and distributing both data and programme resources are constructed at programme initiation, a key lesson we learned from SEA-PHAGES programme development.

## Conclusions

In summary, bacteriophage discovery and genomics is a terrific example of an iREC programme that has excited and engaged thousands of young students. The challenge to science educators now is to find other fields that are similarly well suited, and to implement them at large scale. The initial costs of establishing iREC programmes can be considerable, but the benefits are substantial in terms of student gains, stimulus of faculty career development and the sheer output of research findings. The return on the investment is huge, and we hope that funders and foundations will endorse and support the general advancement of iRECs.
